# Antibacterial effect of silver (I) carbohydrate complexes on oral pathogenic key species in vitro

**DOI:** 10.1186/s12903-016-0201-4

**Published:** 2016-03-23

**Authors:** Markus Reise, Michael Gottschaldt, Carina Matz, Andrea Völpel, Klaus D. Jandt, Ulrich S. Schubert, Bernd W. Sigusch

**Affiliations:** Department of Conservative Dentistry and Periodontology, Jena University Hospital, An der Alten Post 4, 07743 Jena, Germany; Laboratory of Organic and Macromolecular Chemistry, Friedrich Schiller University Jena, Humboldtstr. 10, 07743 Jena, Germany; Jena Center for Soft Matter (JCSM), Philosophenweg 7, 07743 Jena, Germany; Chair of Materials Science, Otto Schott Institute for Materials Research, Friedrich-Schiller-University Jena, Fraunhoferstraße 6, 07743 Jena, Germany

**Keywords:** Silver complexes, Silver nitrate, Antibacterial efficiency, Fibroblasts

## Abstract

**Background:**

It was the aim of this study to evaluate the antibacterial impact of two silver(I) carbohydrate complexes with tripodal thioglycosides, namely tris[2-(β-D-thio-glucopyranosyl)ethyl]-amine-silver(I)-nitrate (**3**) and tris[2-(α-D-thio-manno-pyranosyl)ethyl]-amine-silver(I)-nitrate (**4**), on five oral pathogenic bacterial strains. Furthermore, cytocompatibility was tested using human gingival fibroblasts (HGF).

**Methods:**

Minimum inhibitory concentrations (MIC) were determined on five oral pathogenic bacterial strains by using the broth microdilution method: Fusobacterium nucleatum (ATCC 10953), Aggregatibacter actinomycetemcomitans (ATCC 33384), Porphyromonas gingivalis (ATCC 33277), Streptococcus mutans (ATCC 25175) and Enterococcus faecalis (DSMZ 20376). Furthermore, antimicrobial efficiency was tested using agar diffusion assays. To evaluate cytocompatibility, human gingival fibroblasts (HGFs) were exposed to AgNO_3_ and complex **3** followed by a live/dead staining.

**Results:**

MIC of the silver(I) complexes ranged between 0.625 and 5.0 mmol/L. The silver complexes **3** and **4** showed higher antibacterial efficiency against all tested species than AgNO_3_. Antibacterial efficiency of complexes **3** and **4** on F. nucleatum (≥18 mm) and A. actinomycetemcomitans (≥23 mm) was more pronounced than against P. gingivalis (≥15 mm). Complex **3** (20 mM) induced the largest inhibition zones (30 to 31 mm) on Gram-negative strains. For Gram-positive strains, the largest inhibition zones were achieved by complex **3** (20 mM/S. mutans: 28 mm, E. faecalis: 18 mm). Complex **3** had a lower cytotoxic impact on HGFs compared to AgNO_3_ by the power of ten.

**Conclusions:**

The findings suggest that silver(I) carbohydrate complexes **3** and **4** might function as novel antimicrobial agents for the treatment of periodontal, carious or endodontic diseases.

## Background

The use of silver and silver complexes in medicine and dentistry has attracted much interest over the last decades although the beneficial antibacterial effect of this precious metal has been known since ancient times [1–3].

Numerous fields of application were described that benefit from the antibacterial, antiviral and antifungal efficiency of silver ions including the treatment of oral infectious diseases [4, 5]. These effects of silver ions are based mainly on three mechanisms of action: First, interaction of silver ions with the bacterial DNA; second, destruction of the cell membrane and third, blocking of essential enzymes causing disruption of electron transport [6, 7]. Therefore, the antimicrobial activity of silver(I) complexes strongly depends on the ligand-exchange ability of the complexes in order to release the coordinated silver(I) ions [8].

Inflammatory periodontal disease and dental caries are the two main reasons for tooth loss. Due to the infectious character of these widespread diseases, antibacterial agents play an important role in the treatment of periodontal and carious lesions [9–11]. In particular with regard to periodontal diseases, several in vitro studies have been published in the literature that describe the antibacterial efficiency of silver containing agents against periodontal species [12]. Also in restorative dentistry, suppression of pathogenic species is a significant step towards an effective therapy. For diagnostic purposes and treatment of caries and pulpitis [13], different silver-containing materials were described in the literature including dental adhesives and primers [14, 15], root canal sealers [16] and silver-loaded composite filling materials [17, 18].

Metal complexes substituted with carbohydrates are also increasingly studied for biomedical applications [19]. In 2006, silver(I) complexes based on tripodal thioglycosides were synthesized and analyzed in vitro by our group [20]. Despite the promising results, no clinical applications have been performed so far. One of the main advantages observed, besides their water solubility and stability in solution, was the higher cytocompatibility of the obtained complexes compared to their appropriate silver salts. Those silver complexes had significantly lower cytotoxic and antiproliferative effects [20]. It could also be shown that the tested silver(I) carbohydrate complexes had a broad antibacterial spectrum against different bacterial species. However, so far, these tests have not considered important oral pathogenic species that are associated with periodontal, carious and endodontic infections.

With this background, it was the aim of this study to determine the antimicrobial efficiency of two specific carbohydrate based silver(I) complexes, tris[2-(β-D-thio-glucopyranosyl)ethyl]-amine-silver(I)-nitrate (**3**) and tris[2-(α-D-thio-manno-pyranosyl)ethyl]-amine-silver(I)-nitrate (**4**), on oral pathogenic species. Additionally, we aimed to compare cytocompatibility of the silver(I) complexes and silver nitrate on human gingival fibroblasts.

## Methods

### Silver (I) complexes

**Fig. 1:**
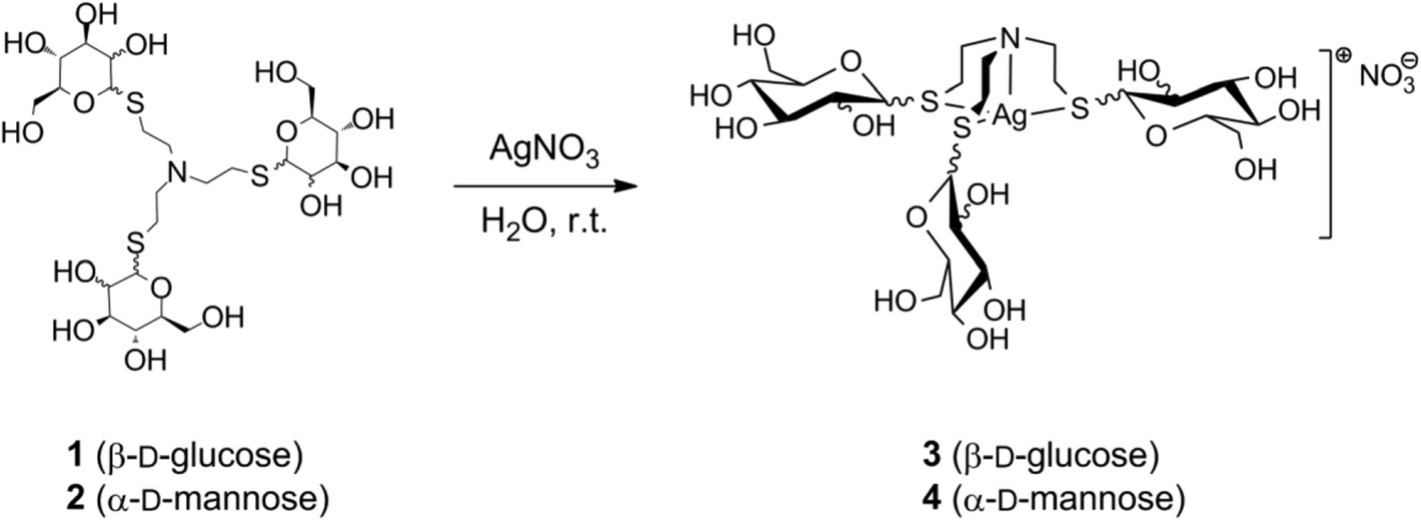
Schematic representation of the synthesis of the studied silver(I) complexes tris[2-(β-d-thio-glucopyranosyl)ethyl]-amine-silver(I)-nitrate (**3**) and tris[2-(α-d-thio-manno-pyranosyl)ethyl]-amine-silver(I)-nitrate (**4**)

The detailed description of the synthesis of the complexes was given earlier [20] and is schematically shown in Fig. 1. In brief, tris[2-(β-D-thio-glucopyranosyl-)ethyl]-amine-silver(I)-nitrate (**3**) was prepared by dissolving 277 mg (0.405 mmol) of the unprotected ligand (**1**) and 68.5 mg (0.405 mmol) AgNO_3_ in water (5 mL) and stirring the solution overnight. After filtration the solvent was evaporated. By diffusion of acetone into a aqueous solution of the raw product 310 mg (89 %) of colorless needles were obtained. ^1^H NMR (400 MHz, D_2_O, DSS): 4.71 (d, 3H, H-1, *J*_1,2_ = 9.8 Hz), 3.31 (t, 3H, H-2, *J*_2,3_ = 9.3 Hz), 3.40 (t, 3H, H-3, *J*_3,4_ = 9.5 Hz), 3.47 - 3.52 (m, 6H, H-4, H-5), 3.96 (d, 3H, H-6, *J*_6,6′_ = 12.2 Hz), 3.72 (dd, 3H, H-6′, *J*_5,6′_ = 6.1 Hz), 3.25 (d, 3H, C*H*, *J* = 12.2 Hz), 3.06 (d, 3H, C*H*, *J* = 15.1 Hz), 2.84-2.99 (m, 3H, C*H*), 2.61 (d, 3H, C*H*, *J* = 13.7 Hz). ^13^C NMR (D_2_O, DSS): 88.88 (*C*1), 82.63 (*C*2), 79.51 (*C*3), 75.38 (*C*4), 71.85 (*C*5), 63.53 (*C*6), 53.30 (N-*C*H_2_-), 34.57 (S-*C*H_2_-). ESI-MS: *m/z* (%): 792.16 (100) (8)^+^, 630.09 (60) [M-C_6_H_10_O_5_]^+^, 468.03 (70) [M-2(C_6_H_10_O_5_)]^+^, 305.97 (95) [M-3(C_6_H_10_O_5_)]^+^. Anal. Calcd for C_24_H_45_AgN_2_O_18_S_3_: C, 33.77; H, 5.31; N, 3.28. Found: C, 32.90; H, 5.68; N, 3.07 %. Complex **4** was prepared analogous starting from the d-mannose derived ligand **2**.

### Bacterial strains

Five oral pathogenic bacterial species were used for this study. Gram-negative strains: Fusobacterium nucleatum (ATCC 10953), Aggregatibacter actinomycetemcomitans (NCTC 9710/DSM 8324) and Porphyromonas gingivalis (ATCC 33277). Gram-positive strains: Streptococcus mutans (ATCC 25175) and Enterococcus faecalis (DSMZ 20376). The bacterial strains were cultivated for 24 h at 37 °C under appropriate (aerobic/anaerobic) conditions in nutrient solution (SCHAEDLER-Broth; Oxoid, Germany) enriched with vitamin K (Roche, Germany). In the next step, the suspensions of bacteria in the logarithmic growth phase were centrifuged (4000 rpm, 5 min/Sigma, Eppendorf, Germany), rinsed twice using PBS (phosphate buffered saline/GIBCO, Germany) and resuspended to obtain a bacterial density of 10^8^ bacteria/mL, that equals an optical density of 0.1 at 640 nm.

### Antibacterial efficiency

#### MIC/MBC

To assess the susceptibility of oral pathogenic bacteria to silver(I) complexes, minimum inhibitory concentrations (MIC) and minimal bactericidal concentrations (MBC) were determined. For this purpose, test agents were dissolved in SCHAEDLER medium (Oxoid, Germany) to obtain a concentration of 40 mM. Serial dilution was performed for all compound solutions. Afterwards, 100 μL of each bacterial suspension (see 2.2) were pipetted into wells of microtiter plates (Greiner, Germany) containing 100 μL of the corresponding diluted compound solution. After incubation at 37 °C for 24 h, the bacterial growth was evaluated. The MIC represents the dilution stage in which no clouding of the test specimen was observed. Pure SCHAEDLER medium served as positive control, SCHAEDLER medium including the same amount of the bacterial suspension as negative control. To distinguish MCI from MBC, 100 μL of each compound solution with concentrations below MIC were spread onto a Petri dish with SCHAEDLER agar (Oxoid, Germany) and cultivated under identical conditions. The concentration of the dilution series where no growth of bacteria was observed was considered as MBC.

#### Agar diffusion tests

To evaluate the antimicrobial concentration-related efficiency of the silver(I) complexes, agar diffusion tests were performed. The general procedure of this test was described previously in the literature [21]. Briefly: 100 μL of each bacterial suspension (10^8^ bacteria/mL) were pipetted and spread onto a Petri dish with Schaedler agar (Oxoid, Germany). 100 μL of each test fluid were then filled into a central hole (diameter: 8 mm) of each Petri dish. After bacteria specific incubation time (>48 h), the diameters of the inhibition zones were measured. For positive (negative) control chlorhexidine (distilled water) was used. Six specimens were prepared for each test fluid (AgNO_3_; complex **3** and **4**) with two different concentrations (10 mM and 20 mM).

### Cytocompatibility

In vitro cytocompatibility tests were performed by exposing the test agents to human gingival fibroblasts (HGFs). These fibroblasts were obtained from a gingiva biopsy of a periodontal healthy female patient (aged 42) by explant method. A written informed consent form was signed by the patient. Beforehand the study was approved by the Ethics Committee Jena (#1881-10/06; Ethics Committee of the Friedrich-Schiller-University Jena at the Medical Faculty, Bachstrasse 18, 07740 Jena, Germany). HGF were chosen since they play a very important role in collagen metabolism and wound healing processes in periodontal lesions.

The cells were cultivated at 37 °C with 5 % of CO_2_ using DMEM (Dulbecco’s Modified Eagle’s Medium) with 10 % of fetal calf serum and 0.1 % of AAS (antibiotic antimycotic solution). In our earlier publication [20] could be demonstrated that cytotoxicity and antiproliferative effects are not related to the type of sugar substituted to the complex. Therefore, only complex **3** was included in cytocompatibility tests of the present study.

HGFs were applied onto sterile cover glasses with a density of 8000 cells/cm^2^ and cultivated for 24 h under culture conditions. Complex **3**, its corresponding ligand **1** and AgNO_3_ were diluted in DMEM to obtain a concentration of 20 mM for each test agent. Afterwards decimal serial dilution was conducted to obtain final concentrations of 20 mM, 2.0 mM, 0.2 mM, 0.02 mM and 0.002 mM. In the next step, the cell medium was removed from the cover glasses and 1 mL of each diluted test agent was applied to the HGFs. Pure cell medium served as negative control. After an incubation time of 24 h, the cell medium and test agent were pipetted off and HGFs were stained using a live/dead colorant (12 mL of PBS + 2 μL of fluorescein diacetate (vital) + 16 μL ethidium bromide [EtBr] (dead)) and evaluated using a Labophot microscope (Nikon, Japan) equipped with a 10xphase contrast objective (λ_ex_ = 455 to 495 nm).

### Statistical analysis

**Fig. 2:**
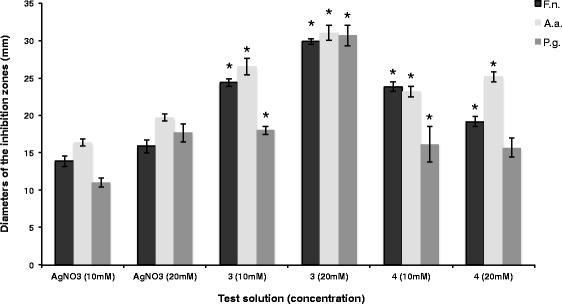
Diameters and standard deviations of the inhibition zones caused by AgNO_3_, silver complexes **3** and **4** for three tested Gram-negative bacterial species. No antibacterial effect was produced by the free ligands **1** and **2**. Statistically significant differences compared to AgNO_3_ (same concentration) are marked (*)

**Fig. 3:**
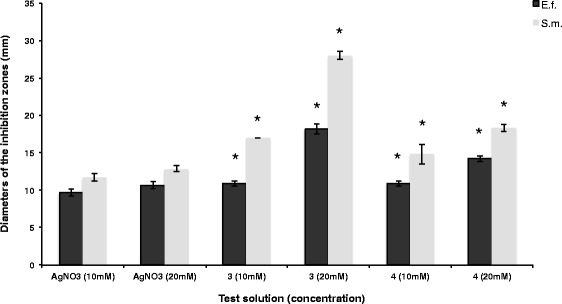
Diameters and standard deviations of the inhibition zones caused by AgNO_3_, the silver complexes **3** and **4** for two tested Gram-positive bacterial species. No antibacterial effect was produced by the free ligands **1** and **2**. Statistically significant differences compared to AgNO_3_ (same concentration) are marked (*)

A t-test was performed using SPSS software (IBM; version 19) to determine whether the differences between inhibition zones of complex **3** and **4** compared to the control (AgNO_3_) are significant. *P* values < 0.005 are considered significant. The bars in the diagrams (Figs. 2 and 3) in which a statistically impact of the silver complex compared to AgNO_3_ at the same concentration (10 mM or 20 mM) was found (*p* < 0.005) were marked (*).

## Results

The antibacterial efficiency of silver(I) carbohydrate complexes (GlcS-)_3_-N AgNO_3_ (**3**) and (ManS-)_3_-N AgNO_3_ (**4**) against the tested bacterial strains Fusobacterium nucleatum, Aggregatibacter actinomycetemcomitans, Porphyromonas gingivalis, Streptococcus mutans and Enterococcus faecalis was more pronounced than the effect of AgNO_3_.

### MIC/MBC

The obtained MIC/MBC values of complexes **3**, **4,** AgNO_3_ and free ligands for the selected bacterial strains are shown in Table 1. For all tested bacterial species, inhibition of growth was observed by using silver complexes and AgNO_3_. Gram-negative bacterial strains had similar concentrations (MICs: P. gingivalis: 0.625 mM [**3**], 1.25 mM [**4**]; A. actinomycetemcomitans: 0.625 mM [**3, 4**] and F. nucleatum: 0.625 mM [**3, 4**]) compared to AgNO_3_ (P. gingivalis: 0.625 mM, A. actinomycetemcomitans: 0.625 mM and F. nucleatum: 0.625 mM). It is apparent that for Gram-positive species higher concentrations of the silver complexes (S. mutans: 10 mM [**3**], 20 mM [**4**] and E. faecalis: 10 mM [**3**], 20 mM [**4**]) are necessary to achieve effective inhibition of bacterial growth (MIC). For a complete suppression of bacteria (MBC), higher concentrations of the tested agents were essential in general. Also in this case a distinct difference between Gram-negative (1.25 to 2.5 mM) and Gram-positive (10 to 20 mM for complexes **3** and **4**) species was observed. No antimicrobial effect was observed for the free ligands **1** and **2**.

**Table 1:** Minimum inhibitory concentrations (MIC) and minimal bactericidal concentrations (MBC) of the tested compounds on oral pathogenic bacterial strains

Bacterial species	Tested agent	Concentration (mM)							
		20	10	5	2.5	1.25	0.625	0.312	0.156
F. nucleatum	AgNO3	–	–	–		–	–	+	+
	3	–	–	–	–	–	– (+)	+	+
	1	+	+	+	+	+	+	+	+
	4	–	–	–	–	–	– (+)	+	+
	2	+	+	+	+	+	+	+	+
A. actinomycetem-comitans	AgNO3	–	–	–	–	–	–	+	+
	3	–	–	–	–	–	– (+)	+	+
	1	+	+	+	+	+	+	+	+
	4	–	–	–	–	–	– (+)	+	+
	2	+	+	+	+	+	+	+	+
P. gingivalis	AgNO3	–	–	–	–	–	– (+)	+	+
	3	–	–	–	–	– (+)	–	+	+
	1	+	+	+	+	+	+	+	+
	4	–	–	–	–	– (+)	+	+	+
	2	+	+	+	+	+	+	+	+
S. mutans	AgNO3	–	–	–	– (+)	– (+)	+	+	+
	3	–	–	– (+)	– (+)	+	+	+	+
	1	+	+	+	+	+	+	+	+
	4	–	– (+)	– (+)	– (+)	– (+)	+	+	+
	2	+	+	+	+	+	+	+	+
E. faecalis	AgNO3	–	–	–	–	+	+	+	+
	3	–	–	– (+)	+	+	+	+	+
	1	+	+	+	+	+	+	+	+
	4	–	– (+)	– (+)	+	+	+	+	+
	2	+	+	+	+	+	+	+	+

+ = Growth of bacteria, (+) = Growth after applying on agar Petri-dishes

- = No growth of bacteria

### Agar diffusion assay

This test showed that silver complexes **3** and **4** inhibit the growth of the tested oral pathogenic bacterial strains. Diameters of the inhibition zones and standard deviations are shown in Fig. 2 (Gram-negative) and 3 (Gram-positive bacterial strains).

For all tested Gram-negative bacterial strains (Fig. 2) silver complexes **3** and **4** generated larger diameters of inhibition zones (10 mM: 18 to 27 mm/20 mM: 16 to 31 mm) compared to AgNO_3_ (10 mM: 11 to 16 mm/20 mM: 16 to 20 mm). The largest inhibition zones were found for complex **3**. It is apparent that in most cases the antibacterial efficiency against F. nucleatum and A. actinomycetemcomitans was more pronounced than against P. gingivalis.

In general, smaller inhibition zones were found for Gram-positive bacterial strains (Fig. 3). It has been shown that all tested agents have a greater antibacterial impact on S. mutans compared to E. faecalis. The largest inhibition zones were achieved by complex **3** (20 mM/S. mutans: 28 mm, E. faecalis: 18 mm).

### Cytocompatibility tests

**Fig. 4:**
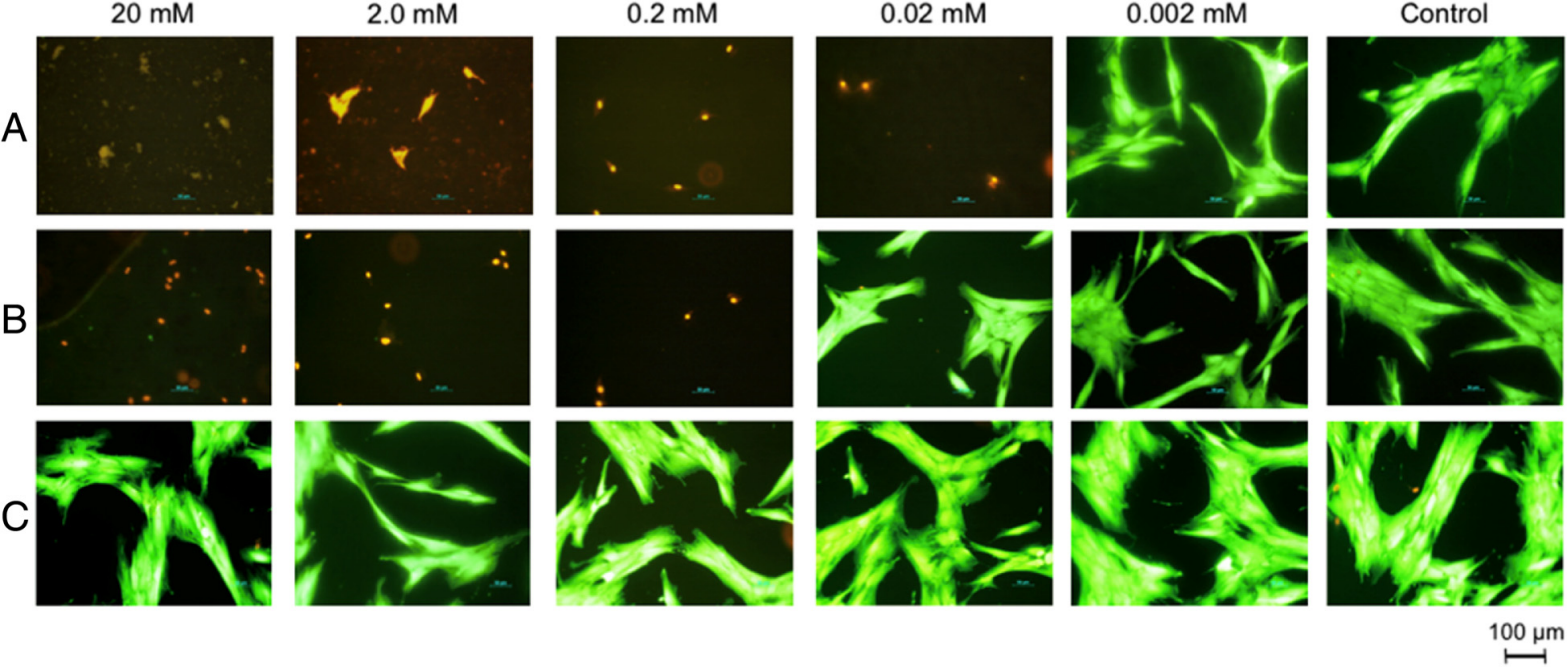
Human gingival fibroblasts (HGFs) after exposure to **a** silver nitrate, **b** silver complex **3** and **c** its corresponding free carbohydrate ligand **1** in different concentrations. Due to live/dead staining vital cells appear green while dead cells are red

Figure 4 shows representative images of the microscopic analysis of the human gingival fibroblasts after being exposed to silver complex **3**, ligand **1** and AgNO_3_. It was noted that AgNO_3_ has a cytotoxic impact on HGFs at a concentration above 0.02 mM. For silver complex **3**, reduced cytotoxicity by the power of ten was shown. No cytotoxic impact on HGFs at all was observed for the free ligand **1**.

## Discussion

Antiinfectious therapeutic agents play an important role in almost every field of medicine. Besides the widespread group of systemically applied classical antibiotics, alternative agents with antibacterial properties are increasingly discussed in the literature. Metal complexes are a promising class of materials with antimicrobial properties. By substitution with sugar residues, metal complexes can be modified to obtain higher levels of solubility and cytocompatibility, which is essential for their use in medicine [19, 22]. Similar silver(I) carbohydrate complexes with tripodal thioglycosides were synthesized and described in 2006 by Gottschaldt et al. [20]. In order to evaluate their possible application in dentistry, i.e. in periodontal, carious and endodontic treatment procedures, two silver complexes were investigated in this in vitro *study:* Tris[2-(β-D-thio-glucopyranosyl)ethyl]-amine-silver(I)-nitrate (**3**) and tris[2-(α-D-thio-manno-pyranosyl)ethyl]-amine-silver(I)-nitrate (**4**).

Fusobacterium nucleatum, Aggregatibacter actinomycetemcomitans and Porphyromonas gingivalis were used in this study as they play an important role in the etiology of periodontal diseases [23–25]. Streptococcus mutans was included due to its strong association with dental caries [18, 26]. Enterococcus faecalis, a representative species of the gastrointestinal flora, was also considered as it is the most frequently isolated bacterial strain in re-infected and insufficiently filled root canals [27, 28].

Relating to the inhibition zones of the agar diffusion assays the novel silver(I) carbohydrate complexes **3** and **4** were able to suppress the growth of all tested Gram-positive and Gram-negative bacterial species (Figs. 2 and 3) adequately in vitro*.* However, the results of the agar diffusion assays should be interpreted for each test compound separately, depending on the ability of the complexes and AgNO_3_ to diffuse through the agarose gel.

MIC/MBC values (Tab. 1) showed that the Gram-negative species F. nucleatum, A. actinomycetemcomitans and P. gingivalis are more susceptible to the silver complexes compared to the Gram-positive species S. mutans and E. faecalis. Similar results were reported previously underlining the higher efficiency of silver ions against the Gram-negative bacterial species A. actinomycetemcomitans, P. gingivalis P. intermedia and E. corrodens [29, 30]. It is assumed that the superior behavior against Gram-negative strains is caused by the lack of the thick protective peptidoglycan layer that can be found in Gram-positive bacterial species [31].

In fields of periodontal therapy there is still need for optimal antiinfectious agents [32]. It is well known that for the treatment of periodontitis the mechanical removal of subgingival biofilms by scaling and root planing is more effective when combined with systemic or local application of antibiotic agents [33, 34]. To avoid side effects and drug resistances caused by high doses of systemically applied antibiotics [35], antimicrobial agents that are applied directly in the periodontal pocket have attracted increasing interest over the last years [21].

The adequate antibacterial effectiveness of silver against periodontal pathogenic species was also shown in several studies. Kawahara et al. [29] demonstrated successfully the antibacterial effect of silver-zeolite against oral pathogenic species under anaerobic conditions to mimic the milieu of periodontal pockets. The study of Lu et al. [36] also focused on the antibacterial impact of differentially sized silver nanoparticles against anaerobic bacteria. In this case, the smallest tested nanoparticles (5 nm) showed higher antimicrobial effects compared to larger particles (15 to 55 nm). This suggests that also the small size of complexes **3** and **4** is a relevant factor for the adequate antibacterial properties. Also the weak binding of silver to thio-ether functions in complexes **3** and **4** leads to their high antibacterial activity. Bacterial proteins function as sulfur donor ligands that replace the ligands of the silver complexes [20, 37].

The high antibacterial efficiency in vitro indicates that the tested silver complexes **3** and **4** might be used as beneficial active ingredients in various types of dental materials. Since the early 20^th^ century, the caries arresting effectiveness of silver nitrate, silver fluoride and silver diammine fluoride has been reported in numerous studies [38–40]. It should be noted that in some studies, black discolorations were observed using silver compounds like silver fluoride in high concentrations (up to 40 %) [39, 41]. In the context of our in vitro investigations no black staining could be detected when the silver complexes were in contact with DMEM medium or during agar diffusion assays. However, in the course of continuative studies (in vitro, later in vivo) we will clarify if silver complexes **3** and **4** also cause black discolorations in appropriate concentrations.

Other studies focused on the modification of increasingly applied dental composite filling materials [17, 18]. In 1999, Yoshida et al. reported on TEGDMA-UDMA based resin composites containing 5 and 7 wt-% silver supported active components, showing their antibacterial efficiency against S. mutans for six months in water while the mechanical qualities of the materials were not negatively affected [16]. With the modification of silver by synthesizing complexes like those used for this study, especially regarding functional groups and hydrophilic properties, material qualities of silver containing resin-based composites might be optimized while maintaining the antibacterial efficiency. Another beneficial aspect is the anti-biofilm effect of silver that was shown in numerous studies [18, 39, 42]. The suppression of bacterial biofilms on surfaces of implants or dental filling materials attracted much interest over the last years as negative effects like periodontitis, periimplantitis, gingivitis and secondary caries can be reduced [43, 44]. Further in vitro investigations of silver complexes should, therefore, also focus on their influence on dental plaque. It should be noted, however, that bacterial biofilms require considerably higher concentrations of antibacterial compounds for an effective removal [45]. Just like for classical antibiotics, the tested silver complexes could not be used in excessive concentrations to avoid cytotoxic side effects.

For E. faecalis, the diameters of the inhibition zones resulting from complex **3** and control AgNO_3_ in lower concentration (10 mM) are relatively small (10 to 11 mm). However, a clinical antibacterial benefit against E. faecalis will most likely by achieved by complex **3** at concentrations above 10 mM (Fig. 3). Since primarily infected endodontic lesions posses a mixture of Gram-positive and Gram-negative bacterial species, disinfection of root canals might also be another field of application in which the silver-complexes could possibly be used. Besides mechanical preparation of the root canals, chemical agents are required for the debridement of infected root dentine. Next to the most common irrigation agents like sodium hypochlorite (NaClO) [46], in addition solutions containing silver complexes might enhance chemical eradication of the pathogen microorganisms. In 2008, Kreth et al. [16] reported about the successful impregnation of silver ions into endodontic sealers to achieve a more profound antibacterial impact. Such approaches should be considered in further studies to thwart the residual bacteria in root canals, which may have resulted in reinfection and treatment failure. The good water solubility of the complexes **3** and **4** might also enable their incorporation in well-established irrigation agents as mentioned above.

The experiments show that complex **3** reveals a lower cytotoxic impact compared to AgNO_3_ on human gingival fibroblasts (HGF). HGF represent the predominant cell type in the oral cavity. Particularly in the context of wound healing in periodontal lesions, HGF play a very important role due to their contribution in collagen metabolism. On this account HGF were chosen for cytocompatibility investigations by our group and in other studies [47, 48].

As shown before in a previous report, cytotoxicity and antiproliferative effects are not related to the type of sugar substituted to the complex [20]. Therefore, only complex **3** was included in cytocompatibility tests of the present study. However, the results of this study are consistent with the cytotoxicity test performed with other cell lines, L-929 (mouse fibroblasts) and HeLa (human cervix carcinoma) in which silver complexes had a significantly lower cytotoxic potential compared to AgNO_3_ [20]. On the other hand, the minimal inhibitory concentrations (see Table 1) of the tested bacterial strains are considerably higher than the concentration of complex **3** (0.02 mM) in which no cytotoxic impact on human gingival fibroblast was found (Fig. 4). This might limit the spectrum of clinical applications. In the treatment of periodontal lesions, active compounds such as antibiotics are applied directly in the periodontal pocket using drug carrier systems for a controlled release [49]. Also in endodontic lesions, the cavity itself limits the field of action, so that direct contact with keratinocytes and other cells is the oral cavity is very unlikely especially when a dental rubber dam is applied.

The widespread use of silver, silver nano particles and silver complexes in medical devices indicate adequate biocompatibility. In a study of Zhao et al. [50], the cytotoxic impact of silver nanoparticles on rat osteoblasts has been reduced by controlling the release rate of silver from titania nanotubes. Also for the tested silver complexes in this study, a controlled release would be essential for their possible application in vivo to balance the ratio between antibacterial efficiency and cytocompatibility.

## Conclusion

The highly water soluble silver(I) carbohydrate complexes (GlcS-)_3_-N AgNO_3_ (**3**) and (ManS-)_3_-N AgNO_3_ (**4**) have a greater antibacterial efficiency against the tested bacterial strains Fusobacterium nucleatum, Aggregatibacter actinomycetemcomitans, Porphyromonas gingivalis, Streptococcus mutans and Enterococcus faecalis than AgNO_3_. Compared to silver nitrate, silver (I) complex **3** possesses a lower cytotoxic effect on human gingival fibroblasts. The results of this study suggest that the innovative silver (I) complexes **3** and **4** represent new potential antibacterial agents for use in various treatment procedures of periodontal, carious and endodontic diseases.

## Abbreviations

AAS: Antibiotic antimycotic solution
A. actino-mycetemcomitans: Aggregatibacter actinomycetemcomitans
DMEM: Dulbecco’s modified eagle’s medium
DNA: Deoxyribonucleic acid
E. faecalis: Enterococcus faecalis
F. nucleatum: Fusobacterium nucleatum
HGF: Human gingival fibroblasts
MIC: Minimum inhibitory concentrations
P. gingivalis: Porphyromonas gingivalis
S. mutans: Streptococcus mutans
